# Improved post-transplant outcomes for elderly acute myeloid leukemia patients conditioned with FLU/BU4 rather than conventional MAC regimens

**DOI:** 10.1038/s41409-025-02573-7

**Published:** 2025-04-05

**Authors:** Takuya Shimizu, Yasuyuki Arai, Tadakazu Kondo, Shingo Yano, Yoshimitsu Shimomura, Noriko Doki, Takahiro Fukuda, Tetsuya Nishida, Satoshi Takahashi, Shuichi Ota, Yoshinobu Kanda, Takuro Kuriyama, Naoki Kurita, Toshiro Kawakita, Yuta Hasegawa, Nobuhiro Hiramoto, Makoto Onizuka, Yoshiko Atsuta, Masamitsu Yanada

**Affiliations:** 1 Department of Hematology, Kyoto University Hospital, Kyoto, Japan; 2 Department of Hematology, Kobe City Hospital Organization Kobe City Medical Center General Hospital, Hyogo, Japan; 3 Division of Clinical Oncology and Hematology, The Jikei University School of Medicine, Tokyo, Japan; 4 Hematology Division, Tokyo Metropolitan Cancer and Infectious Diseases Center Komagome Hospital, Tokyo, Japan; 5 Department of Hematopoietic Stem Cell Transplantation, National Cancer Center Hospital, Tokyo, Japan; 6 Deparment of Hematology, Japanese Red Cross Aichi Medical Center Nagoya Daiichi Hospital, Aichi, Japan; 7 Department of Hematology/Oncology, The Institute of Medical Science, The University of Tokyo, Tokyo, Japan; 8 Department of Hematology, Sapporo Hokuyu Hospital, Hokkaido, Japan; 9 Division of Hematology, Jichi Medical University Saitama Medical Center, Saitama, Japan; 10 Department of Hematology, Hamanomachi Hospital, Fukuoka, Japan; 11 Department of Hematology, University of Tsukuba Hospital, Ibaraki, Japan; 12 Department of Hematology, NHO Kumamoto Medical Center, Kumamoto, Japan; 13 Department of Hematology, Hokkaido University Hospital, Hokkaido, Japan; 14 Department of Hematology/Oncology, Tokai University School of Medicine, Kanagawa, Japan; 15 Japanese Data Center for Hematopoietic Cell Transplantation, Aichi, Japan; 16 Department of Registry Science for Transplant and Cellular Therapy, Aichi Medical University School of Medicine, Aichi, Japan; 17 Department of Hematology and Oncology, Nagoya City University East Medical Center, Aichi, Japan

**Keywords:** Haematopoietic stem cells, Risk factors

## Abstract

It is uncertain whether FLU/BU4 regimens, classified as myeloablative conditioning (MAC), improve prognosis compared to conventional MAC regimens (conv-MAC) such as CY/TBI and BU/CY. We compared FLU/BU4 with conv-MAC among 6551 patients (FLU/BU4 905, conv-MAC 5646), including acute myeloid leukemia (AML) patients aged 16–59 who received a first allogeneic transplantation from the Japanese nationwide registry. The primary endpoint was overall survival (OS), while secondary endpoints were treatment-related mortality (TRM) and relapse at 3 years. Results indicated comparable OS for conv-MAC regimens among the entire cohort (3-year OS: FLU/BU4 50.4% vs. conv-MAC 55.4%, *p* < 0.001). Subgroup analysis focusing on elderly patients (aged 50–59) indicated that FLU/BU4 showed a statistically significant improvement in OS (47.0% vs. 42.8%, *p* = 0.036). Notably, for patients in this cohort transplanted at complete remission (CR), FLU/BU4 demonstrated a substantial advantage over conv-MAC with superior OS (HR 0.75, *p* = 0.046), lower TRM (HR 0.66, *p* = 0.035), and comparable relapse (HR 0.84, *p* = 0.390). These benefits were not observed in elderly patients transplanted at non-CR or in other age groups. In summary, our findings suggest that FLU/BU4 regimen, rather than conv-MAC, may be preferable in MAC-tolerant AML patients, aged 50–59 at CR status. This treatment improves survival by reducing TRM without increasing relapse risk.

## Introduction

Allogeneic hematopoietic stem cell transplantation (allo-HSCT) is effective in patients with acute myeloid leukemia (AML). In terms of conditioning, conventional myeloablative conditioning (MAC) regimens, including cyclophosphamide plus total-body irradiation (CY/TBI) or cyclophosphamide plus busulfan (BU/CY) are commonly used. To strengthen the anti-tumor effect, intensive MAC regimens, which added cytarabine or etoposide to CY/TBI, have also been studied in high-risk patients in relation to relapse [1].

However, it remains to be seen how intensity of conditioning regimens can be reduced without increasing relapse- or treatment-related mortality (TRM). One possible regimen is fludarabine (FLU) plus BU (FLU/BU4), which is classified as MAC, according to criteria of the Center for International Blood and Marrow Transplant Research (CIBMTR), because the total dosage of BU is above 9.6 mg/kg [2]. However, comparisons of FLU/BU4 with conventional MAC (conv-MAC), including BU/CY and CY/TBI regimens have not been adequately investigated. A decrease of TRM in FLU/BU4 has been observed in direct comparison with BU/CY in the whole cohort with wide range of ages [3] or in patients 51 years and older [4]. However, it is still unknown which patients benefit most from FLU/BU4 compared to conv-MAC regimens. A phase 3 study comparing Bu/Cy vs. Flu/Bu in leukemia and MDS patients determined that non-relapse mortality was comparable, but OS was better in BuCy [5].

We hypothesized that an FLU/BU4 regimen can improve overall survival (OS) in all or some subgroups of elderly patients. Thus, we conducted a retrospective cohort study using the Japanese nationwide registry, in order to identify the population in which FLU/BU4 regimen should be employed in preference to conv-MAC.

## Materials and methods

### Inclusion and exclusion criteria

Data were obtained from the Japanese nationwide registry for MAC-eligible adult patients (16–59 years at HSCT) with acute myeloid leukemia (AML) who underwent first allogenic HSCT between January 1, 2000, and December 31, 2019.

FLU/BU4 was defined as follows; FLU 150 or 180 mg/m^2^ + BU i.v. 12.8 mg/kg or *p.o*. 16 mg/kg, with or without TBI 2–4 Gy. The conv-MAC group was defined as BU/CY (BU 12.8 mg/kg; CY 120 mg/kg), CY/TBI (CY 120 mg/kg; TBI 10–12 Gy/4–6 fractions), or intensive MAC regimens with cytarabine 8 or 12 g/m^2^, etoposide 30–60 mg/kg, or melphalan 140–180 mg/m^2^ to CY/TBI.

Our protocol complied with the Declaration of Helsinki, and was approved by the Transplant Registry Unified Management Program Data Management and Ethics Committees of Kyoto University, where the study was performed. Written informed consent was obtained from each patient at each institution.

### Data collection and definition of each covariate

Baseline data was extracted from the Japanese nationwide registry. Patient age at HSCT (16–29, 30–49, 50–59 years of age), sex, disease status at HSCT (complete remission [CR] vs non-CR including relapse or primary induction failure), performance status (PS; 0–1 vs 2–4), and hematopoietic cell transplant commodity index (HCT-CI; 0–2 vs 3–29) were included. We also extracted karyotype risk (favorable, intermediate, poor, unevaluable), donor source (related bone marrow [BM], related peripheral blood stem cell [PB], unrelated BM, unrelated PB, cord blood), HLA match status (8/8 matched vs. 7/8 or less matched), and graft-versus-host disease (GVHD) prophylaxis (cyclosporin A [CyA] +/− methotrexate [MTX]-based, tacrolimus [Tac] +/− MTX-based). The primary endpoint was overall survival (OS) at 3-years. Secondary endpoints were incidence of relapse and TRM at 3-years.

### Statistical analyses

Patient characteristics were compared by Fisher’s test, χ-square test, or Student’s *t* test. Probabilities for OS were estimated according to the Kaplan–Meier method and groups were compared using log-rank tests. Incidence of relapse and TRM were compared using Gray’s test. Each variable was evaluated by log-rank test. Multivariate analysis was performed using Cox proportional hazard models. Factors with *p* < 0.1 or variables with significance in previous studies were included; conditioning regimen (FLU/BU4 vs. conv-MAC), karyotype risk, GVHD prophylaxis (CyA +/− MTX, Tac +/− MTX), HCT-CI (0–2 vs. 3-), HLA-matched status at 8-locus (GVH direction; full vs. mismatched), donor sources, and PS (0–1 vs. 2–4). In TRM, event of relapse was regarded as competing risk. The Fine-Gray proportional hazard model was used in relapse and TRM analysis. All p values were 2-sided, and *p* < 0.05 was used as the cutoff for statistical significance. All statistical analyses were performed using EZR [6] (Saitama Medical Center, Jichi Medical University, Saitama, Japan), a graphical user interface for the R4.2.1 software program (R Foundation for Statistical Computing, Vienna, Austria) and R studio (version 2022.02.3).

## Results

### Characteristics of patients and transplants

A total of 6551 AML patients who underwent allogenic HSCT with MAC regimens were extracted from the Japanese nationwide registry. Among them, 905 patients received FLU/BU4, while 5646 patients received conv-MAC. Detailed patient characteristics by age group are shown in Table 1. In the whole cohort, patients who received FLU/BU4 were associated with poor karyotype risk, poorer disease status at HSCT, reduced likelihood of CyA and MTX as a GVHD prophylaxis, higher HCI-CI score and worse PS. There was a similar tendency in patients aged 50–59, except that the HCT-CI score was comparable between FLU/BU4 and conv-MAC. HLA mismatch status was irrelevant to conditioning regimens. Conv-MAC was mainly composed of CY/TBI (*N* = 414) and BU/CY (*N* = 428) in this elder cohort.

**Table 1:** Patient characteristics of the entire study population and those aged 50–59.

	Whole cohort			Cohort aged 50–59		
Variables	FLU/BU4 (*N* = 905)	conv-MAC (*N* = 5646)	*p*	FLU/BU4 (*N* = 593)	conv-MAC (*N* = 1062)	*p*
Patient sex			0.745			0.640
Female	390 (43.1%)	2471 (43.8%)		249 (42.0%)	434 (40.9%)	
Male	514 (56.9%)	3175 (56.2%)		343 (57.8%)	628 (59.1%)	
PS at HSCT			<0.001			0.759
0–1	828 (91.5%)	5194 (94.5%)		546 (92.1%)	967 (92.6%)	
2–4	77 (8.5%)	300 (5.5%)		47 (7.9%)	77 (7.4%)	
HCT-CI			<0.001			0.198
0–2	722 (90.2%)	3928 (94.4%)		466 (78.6%)	714 (67.2%)	
3-	78 (9.8%)	232 (5.6%)		53 (8.9%)	63 (5.9%)	
Disease status at HSCT			0.402			0.056
CR	559 (65.9%)	3617 (68.1%)		366 (66.5%)	591 (60.6%)	
non-CR	289 (34.1%)	1694 (31.9%)		184 (33.5%)	384 (39.4%)	
Chromosomal risk			<0.001			0.006
favorable	100 (12.6%)	899 (17.2%)		51 (9.8%)	123 (12.5%)	
intermediate	475 (60.0%)	3010 (57.5%)		322 (61.6%)	587 (59.7%)	
poor	175 (22.1%)	917 (17.5%)		123 (23.5%)	203 (20.7%)	
unevaluable	42 (5.3%)	412 (7.8%)		27 (5.2%)	70 (7.1%)	
Donor source			<0.001			<0.001
related BM	52 (5.8%)	775 (14.0%)		40 (6.8%)	129 (12.5%)	
related PB	295 (32.6%)	1146 (20.7%)		169 (28.6%)	193 (18.6%)	
unrelated BM	363 (40.2%)	2198 (39.7%)		251 (42.5%)	432 (41.7%)	
unrelated PB	40 (4.4%)	92 (1.7%)		27 (4.6%)	15 (1.4%)	
CB	153 (16.9%)	1321 (23.9%)		104 (17.6%)	267 (25.8%)	
HLA mismatch			0.079			0.707
matched	306 (33.8%)	1634 (28.9%)		228 (38.4%)	313 (29.5%)	
mismatched	350 (38.6%)	1604 (28.4%)		209 (35.2%)	302 (28.4%)	
GVHD prophylaxis			<0.001			<0.001
CyA+MTX	211 (23.6%)	2536 (46.0%)		153 (25.8%)	443 (41.7%)	
CyA w/o MTX	27 (3.0%)	96 (1.7%)		14 (2.4%)	19 (1.8%)	
Tac+MTX	450 (50.3%)	2649 (48.0%)		309 (52.1%)	525 (49.5%)	
Tac w/o MTX	206 (23.0%)	233 (4.2%)		111 (18.7%)	45 (4.2%)	
Follow up for survivors (median, days)						
	489	778		416	434.5	

*CR* complete remission, *CyA* cyclosporine, *MTX* methotrexate, *Tac* tacrolimus, *HCT-CI* Hematopoietic cell transplantation-specific comorbidity index, *HLA* human leukocyte antigen, *GVH* graft-versus-host, *PS* performance status, *w/o* without.

### Conv-MAC was associated with better OS in the whole cohort, while FLU/BU4 had superior OS in elderly patients

**Fig. 1:**
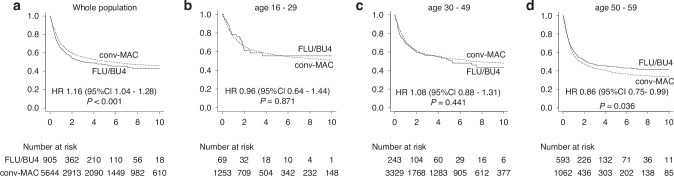
OS between FLU/BU4 and conv-MAC regimens. OS between FLU/BU4 vs. conv-MAC in (**a**) all patients (aged 16–59), **b** patients aged 16–29, **c** patients aged 30–49, and (**d**) patients aged 50–59. OS was compared with univariate analyses using the log-rank method.

In the whole MAC-eligible cohort (aged 16–59), conv-MAC had superior OS (3-year OS, FLU/BU4 vs. conv-MAC; 50.4% [95% confidence interval (CI) 46.8–53.9%] vs. 55.4% [54.0–56.7%], *p* < 0.001; Fig. 1a). For the purpose of subgroup analyses, patients were divided into three groups according to age: a young cohort, 16–29 years (FLU/BU4 69, conv-MAC 1253), middle-aged cohort, 30–49 years (FLU/BU4 243, conv-MAC 3329), and an elderly cohort, 50–59 years (FLU/BU4 593, conv-MAC 1062). Three-year OS was comparable in the young cohort (FLU/BU4 58.6% [95% CI, 44.4–70.3%] vs. conv-MAC 61.0% [58.1–63.8%], *p* = 0.871; Fig. 1b) and middle-aged cohort (FLU/BU4 56.3% [49.3–62.7%] vs. conv-MAC 57.3% [55.5–59.0%], *p* = 0.441; Fig. 1c). However, in the elderly cohort, FLU/BU4 showed improved 3-year OS (FLU/BU4 47.0% [42.6–51.3%] vs. conv-MAC 42.8% [39.7–45.9%], *p* = 0.036; Fig. 1d). Sensitivity analyses comparing FLU/BU4 and each conv-MAC regimen (CY/TBI or BU/CY) indicated the superiority of FLU/BU4 (data not shown).

### FLU/BU4 improved OS in the CR group, but not in the non-CR group at HSCT in elderly patients

**Fig. 2:**
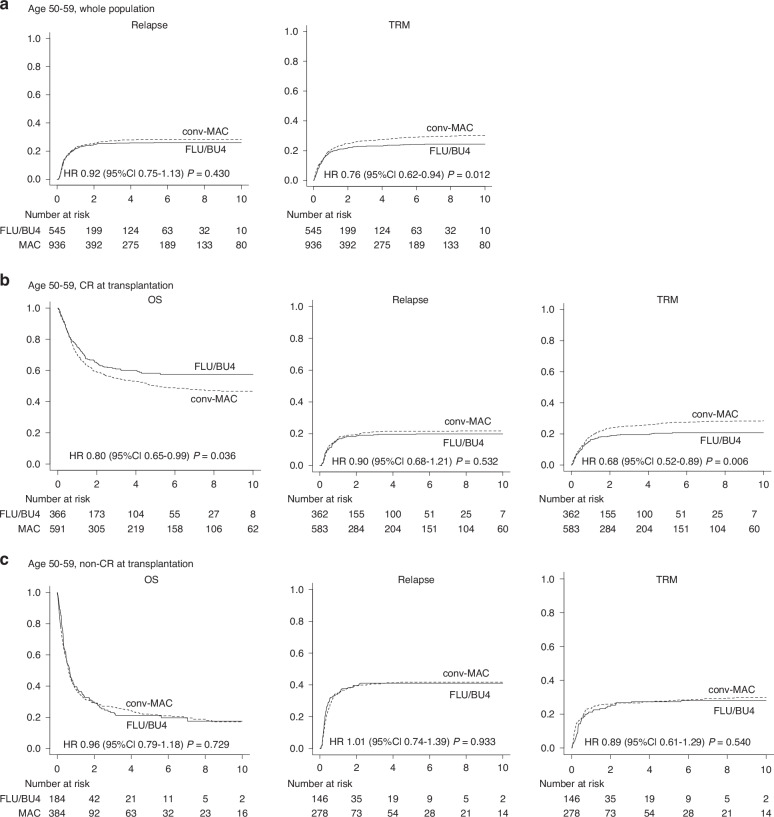
OS, relapse, and TRM in patients aged 50–59. Relapse, relapse, and TRM of (**a**) the cohort of patients aged 50–59. **b** The subgroup of CR at HSCT, and (**c**) The subgroup of non-CR at HSCT.

In the elderly group, incidence of relapse at 3-years was comparable (FLU/BU4 25.5% [95% CI, 21.9–29.2%] vs. conv-MAC 27.4% [24.5–30.2%], *p* = 0.455; Fig. 2a), whereas TRM was significantly favorable in FLU/BU4 (FLU/BU4 22.9% [95% CI, 19.5–26.5%] vs. conv-MAC 26.6% [23.8–29.5%], *p* = 0.020; Fig. 2a). These data suggested improved OS was mainly due to decreased TRM in this group.

Post-transplant relapse and TRM are strongly influenced by disease status at HSCT. Accordingly, patients were divided into CR and non-CR groups. The CR group showed significantly superior OS in the FLU/BU4 group (FLU/BU4 60.9% [95% CI, 55.2–66.1%] vs. conv-MAC 55.0% [50.7–59.0%] at 3 years, *p* = 0.036; Fig. 2b). Incidence of relapse was comparable: FLU/BU4 19.3% [95% CI, 15.4– 23.6%] vs. conv-MAC 21.3% [18.0–24.7%] at 3 years, *p* = 0.532; Fig. 2b), and TRM was significantly lower in FLU/BU4 (FLUBU4 19.6% [95% CI, 15.7–23.9%] vs. conv-MAC 25.0% [21.6–28.6%], *p* = 0.008; Fig. 2b).

On the other hand, among non-CR patients, FLU/BU4 and conv-MAC patients showed superimposed outcomes 3-year OS (FLU/BU4 23.1% [95% CI, 16.8–30.0%] vs. conv-MAC 26.7% [22.2–31.4%] *p* = 0.729; Fig. 2c), incidence of relapse (FLU/BU4 41.1% [95% CI, 33.0–49.0%] vs. conv-MAC 40.6% [34.8–46.4%] *p* = 0.933; Fig. 2c) and TRM (FLU/BU4 26.7% [95% CI, 19.8–34.1%] vs. conv-MAC 26.6% [21.6–31.9%], *p* = 0.626; Fig. 2c). These subgroup analyses of elderly patients revealed that FLU/BU4 showed better OS and TRM without increased relapse, but only in CR patients.

### Multivariate analyses

We next performed multivariate analyses to adjust confounders in selection of conditioning regimens between FLU/BU4 vs. conv-MAC in the elderly cohort. In multivariate analyses, FLU/BU4 (HR 0.84; 95% CI 0.69–1.02, *p* = 0.071), poor risk karyotype (HR 2.08; 95% CI 1.6057–2.737, *p* < 0.001), HLA-mismatched HSCT (HR 1.15; 95% CI 0.92–1.44, *p* = 0.210), and PS 2–4 (HR 2.22; 95% CI 1.54–2.98, *p* < 0.001) were associated with 3-year OS in elderly patients (Table 2). However, the relapse rate and TRM showed no significant difference between FLU/BU4 and conv-MAC.

**Table 2:** Multivariate analyses of OS, relapse, and TRM in patients aged 50–59.

Whole cohort aged 50–59		OS	Relapse	TRM
Variables				
Conditioning	FLU/BU4	0.84 (0.69–1.02, *p* = 0.071)	0.78 (0.58–1.04, *p* = 0.095)	0.83 (0.62–1.12, *p* = 0.220)
Chromosomal risk	intermediate	1.01 (0.78–1.31, *p* = 0.921)	1.38 (0.93–2.04, *p* = 0.110)	1.17 (0.81–1.68, *p* = 0.390)
	poor	2.08 (1.57–2.77, *p* < 0.001)	2.55 (1.61–4.02, *p* < 0.001)	1.45 (0.94–2.25, *p* = 0.095)
	unevaluable	1.14 (0.70–1.83, *p* = 0.604)	1.82 (0.96–3.46, *p* = 0.066)	0.76 (0.33–1.76, *p* = 0.520)
HLA	mismatched	1.15 (0.92–1.44, *p* = 0.210)	0.90 (0.63–1.28, *p* = 0.550)	1.44 (1.04–2.01, *p* = 0.029)
PS	2–4	2.22 (1.65–2.98, *p* < 0.001)	1.39 (0.85–2.29, *p* = 0.190)	1.47 (0.89–2.41, *p* = 0.130)
HCT-CI	3-	0.91 (0.67–1.25, *p* = 0.568)	0.90 (0.55–1.47, *p* = 0.670)	0.83 (0.51–1.35, *p* = 0.450)
Donor source	related-PB	1.42 (0.96–2.12, *p* = 0.082)	0.84 (0.49–1.44, *p* = 0.530)	2.37 (1.16–4.89, *p* = 0.019)
	unrelated-BM	1.28 (0.86–1.90, *p* = 0.219)	0.70 (0.41–1.19, *p* = 0.190)	2.21 (1.06–4.58, *p* = 0.034)
	unrelated-PB	0.89 (0.43–1.85, *p* = 0.760)	0.93 (0.40–2.16, *p* = 0.860)	1.53 (0.48–4.85, *p* = 0.470)
	CB	1.37 (0.90–2.11, *p* = 0.145)	0.79 (0.43–1.45, *p* = 0.450)	2.21 (1.04–4.72, *p* = 0.041)
GVHD prophylaxis	CyA w/o MTX	0.92 (0.40–2.09, *p* = 0.839)	0.62 (0.16–2.38, *p* = 0.490)	1.35 (0.44–4.20, *p* = 0.600)
	Tac + MTX	0.85 (0.67–1.09, *p* = 0.199)	0.95 (0.66–1.38, *p* = 0.800)	0.78 (0.54–1.12, *p* = 0.170)
	Tac w/o MTX	1.01 (0.71–1.41, *p* = 0.975)	0.85 (0.49–1.47, *p* = 0.560)	0.95 (0.56–1.59, *p* = 0.830)
CR cohort aged 50–59				
Conditioning	FLU/BU4	0.75 (0.56–0.99, *p* = 0.046)	0.84 (0.57–1.25, *p* = 0.390)	0.66 (0.45–0.97, *p* = 0.035)
Chromosomal risk	intermediate	1.24 (0.83–1.85, *p* = 0.302)	1.80 (0.96–3.34, *p* = 0.067)	1.23 (0.76–1.98, *p* = 0.400)
	poor	2.07 (1.28–3.32, *p* = 0.003)	4.06 (1.99–8.26, *p* < 0.001)	1.24 (0.68–2.31, *p* = 0.490)
	unevaluable	1.31 (0.65–2.64, *p* = 0.455)	2.23 (0.88–5.67, *p* = 0.091)	0.92 (0.33–2.54, *p* = 0.870)
HL	mismatched	1.16 (0.84–1.61, *p* = 0.374)	0.76 (0.47–1.24, *p* = 0.270)	1.63 (1.08–2.47, *p* = 0.020)
PS	2–4	1.77 (0.97–3.20, *p* = 0.061)	0.87 (0.31–2.47, *p* = 0.800)	1.66 (0.82–3.35, *p* = 0.160)
HCT-CI	3-	0.79 (0.47–1.36, *p* = 0.406)	0.70 (0.31–1.54, *p* = 0.380)	0.88 (0.45–1.71, *p* = 0.700)
Donor source	related-PB	1.96 (1.07–3.59, *p* = 0.030)	0.88 (0.41–1.88, *p* = 0.740)	2.93 (1.10–7.80, *p* = 0.031)
	unrelated-BM	1.81 (0.98–3.33, *p* = 0.056)	0.89 (0.43–1.86, *p* = 0.760)	2.77 (1.01–7.57, *p* = 0.047)
	unrelated-PB	1.43 (0.52–3.92, *p* = 0.484)	1.15 (0.38–3.45, *p* = 0.800)	2.26 (0.54–9.46, *p* = 0.260)
	CB	2.04 (1.03–4.02, *p* = 0.040)	0.96 (0.39–2.32, *p* = 0.920)	2.54 (0.89–7.28, *p* = 0.082)
GVHD prophylaxis	CyA w/o MTX	1.01 (0.31–3.26, *p* = 0.985)	0.60 (0.10–3.70, *p* = 0.580)	1.52 (0.31–7.47, *p* = 0.610)
	Tac + MTX	0.67 (0.47–0.96, *p* = 0.027)	0.79 (0.47–1.31, *p* = 0.360)	0.73 (0.45–1.21, *p* = 0.230)
	Tac w/o MTX	0.85 (0.49–1.47, *p* = 0.564)	0.68 (0.29–1.61, *p* = 0.380)	1.00 (0.50–2.01, *p* = 0.990)
non-CR cohort aged 50–59				
Conditioning	FLU/BU4	1.02 (0.75–1.37, *p* = 0.916)	0.73 (0.44–1.18, *p* = 0.200)	1.42 (0.80–2.51, *p* = 0.230)
Chromosomal risk	intermediate	0.88 (0.57–1.34, *p* = 0.537)	1.11 (0.59–2.09, *p* = 0.750)	1.32 (0.63–2.76, *p* = 0.470)
	poor	1.96 (1.26–3.06, *p* = 0.003)	1.75 (0.82–3.73, *p* = 0.150)	1.61 (0.68–3.80, *p* = 0.280)
	unevaluable	1.12 (0.54–2.32, *p* = 0.771)	1.63 (0.55–4.82, *p* = 0.380)	0.76 (0.15–3.76, *p* = 0.730)
HLA	mismatched	0.99 (0.71–1.40, *p* = 0.967)	0.79 (0.44–1.44, *p* = 0.450)	1.25 (0.62–2.50, *p* = 0.530)
PS	2–4	2.24 (1.52–3.27, *p* < 0.001)	1.58 (0.85–2.92, *p* = 0.140)	1.24 (0.56–2.70, *p* = 0.600)
HCT-CI	3-	1.08 (0.69–1.69, *p* = 0.733)	1.11 (0.55–2.21, *p* = 0.770)	0.81 (0.34–1.89, *p* = 0.620)
Donor source	related-PB	0.98 (0.54–1.76, *p* = 0.946)	0.72 (0.28–1.87, *p* = 0.500)	1.34 (0.40–4.45, *p* = 0.640)
	unrelated-BM	0.99 (0.57–1.73, *p* = 0.990)	0.61 (0.26–1.41, *p* = 0.240)	1.06 (0.33–3.43, *p* = 0.920)
	unrelated-PB	0.75 (0.24–2.28, *p* = 0.607)	0.91 (0.25–3.27, *p* = 0.890)	0.66 (0.08–5.75, *p* = 0.710)
	CB	0.98 (0.55–1.78, *p* = 0.965)	0.61 (0.25–1.54, *p* = 0.300)	1.54 (0.47–5.04, *p* = 0.480)
GVHD prophylaxis	CyA w/o MTX	0.87 (0.20–3.80, *p* = 0.858)	1.50 (0.22–10.1, *p* = 0.680)	0.00 (0.00–0.00, *p* < 0.001)
	Tac + MTX	1.02 (0.71–1.46, *p* = 0.913)	1.30 (0.70–2.39, *p* = 0.400)	0.77 (0.40–1.47, *p* = 0.430)
	Tac w/o MTX	0.99 (0.60–1.63, *p* = 0.959)	1.11 (0.55–2.21, *p* = 0.770)	0.67 (0.26–1.72, *p* = 0.410)

Detailed analyses focusing on remission status at HSCT showed that in CR patients FLU/BU4 (HR 0.75; 95% CI 0.56–0.99, *p* = 0.046), poor risk karyotype (HR 2.07; 95% CI 1.28–3.32, *p* = 0.003), PS 2–4 (HR 1.77; 95% CI 0.97–3.20, *p* = 0.061), unrelated-BM (HR 1.81; 95% CI 0.98–3.33, *p* = 0.056) and GVHD prophylaxis by Tac and MTX (HR 0.67; 95% CI 0.47–0.96, *p* = 0.0027) were significantly associated with 3-year OS, with comparable relapse (HR 0.84; 95% CI 0.57–1.23, *p* = 0.390) and lower TRM (HR 0.66; 95% CI 0.45–0.97, *p* = 0.035) in FLU/BU4 (Table 2). These advantages of FLU/BU4 over conv-MAC were not observed in non-CR older patients (Table 2) or in other age groups (data not shown).

### Incidence of GVHD and causes of decreased TRM in elderly patients

Lastly, we examined adverse events in each cohort. There was no significant difference in the whole cohort of elderly patients for the cumulative incidence of aGVHD grade 2–4 (FLU/BU4 36.6% vs. conv-MAC 32.7%, *p* = 0.156), aGVHD grade 3–4 (FLU/BU4 10.3% vs. conv-MAC 9.8%, *p* = 0.542) or cGVHD (FLU/BU4 34.9% vs. conv-MAC 36.9%, *p* = 0.284) (Table 3).

**Table 3:** Incidence of aGVHD and cGVHD in patients aged 50–59.

Cohort	Variables		Incidence		*p*
			FLU/BU4 (%)	conv-MAC (%)	
Whole Cohort aged 50–59					
	aGVHD	Grade 2–4	36.6 (32.7–40.5)	32.7 (29.9–35.5)	0.156
		Grade 3–4	10.3 (8.0–12.9)	9.8 (8.1–11.7)	0.542
	cGVHD		34.9 (30.5–39.3)	36.9 (33.6–40.1)	0.284
CR cohort aged 50–59					
	aGVHD	Grade 2–4	33.3 (28.5–38.2)	32.7 (28.9–36.5)	0.939
		Grade 3–4	9.0 (6.4–12.2)	9.3 (7.1–11.8)	0.814
	cGVHD		38.5 (32.9–44.0)	40.1 (35.7–44.4)	0.518
non-CR cohort aged 50–59					
	aGVHD	Grade 2–4	40.2 (33.1–47.2)	31.8 (27.2–36.5)	0.063
		Grade 3–4	10.9 (6.9–15.9)	9.9 (7.2–13.1)	0.647
	cGVHD		28.2 (19.7–37.3)	30.7 (25.4–36.2)	0.258

*aGVHD* acute graft versus host disease, *cGVHD* chronic graft versus host disease.

**Fig. 3:**
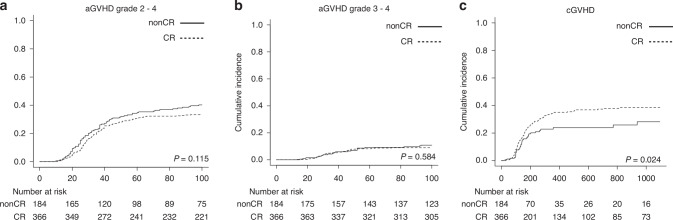
Incidence of aGVHD and cGVHD in patients aged 50–59 who received FLU/BU4 as conditioning. Incidence of (**a**) aGVHD grade 2–4, **b** aGVHD grade 3–4, and (**c**) cGVHD were compared in patients aged 50–59 between CR and non-CR patients. The bold line indicates the non-CR group whereas the dashed line indicates the CR group.

Among elderly CR patients, the incidence of GVHD was comparable between FLU/BU4 and conv-MAC, whereas in non-CR patients, the incidence of aGVHD grade 2–4 was slightly higher in the FLU/BU4 group than in conv-MAC (Table 3). Comparison of incidence in aGVHD or cGVHD between the CR and non-CR cohorts conditioned with a FLU/BU4 regimen are shown in Fig. 3. Significantly higher incidence of cGVHD was observed in the CR cohort. Causes of death among elderly patients according to conditioning regimen are listed in Supplemental Table 1. Fewer patients died of organ failure (heart or lung) or thrombotic microangiopathy.

## Discussion

In this retrospective cohort study using Japanese registry data to compare FLU/BU4 vs. conv-MAC in the MAC-eligible cohort, we reached the following novel conclusions: 1) FLU/BU4 and conv-MAC are associated with comparable OS in younger patients (≤49 years), 2) FLU/BU4 improved OS and TRM without increasing the relapse rate of patients aged 50–59, but this improvement was demonstrated only in CR patients at the time of HCST, whereas non-CR patients showed comparable OS between FLU/BU4 vs. conv-MAC.

Comparisons between FLU/BU4 and conv-MAC indicate that CR patients aged 50–59 can definitely benefit from using an FLU/BU4 regimen; however, our study has some limitations. First, this is the retrospective cohort study using national data in Japan, and these results cannot necessarily be extrapolated to the global population [7]. Future prospective studies are awaited from other countries. Second, the administration method of busulfan has varied according to the decade. Initially, oral busulfan was used. Then it was switched to intravenous busulfan 4 times a day, followed by therapeutic drug monitoring (TDM)-guided busulfan administration and once-daily administration. TDM-guided busulfan administration showed improvement compared to the conventional dosing method, using body weight [8]^.^ Therefore, subgroup analyses focusing on busulfan administration method may be necessary in the future. Sensitivity analysis including only intravenous busulfan indicated similar results regarding the competition between Flu/BU4 vs. conv-MAC (data not shown). Moreover, the year of transplant in not included in the study to build the new prognosis model. Subgroup analyses indicated that FLU/BU4 increased only recently (i.e. after 2010), but it was constantly superior to conv-MAC in each period of year (data not shown).

FLU/BU4 is already established as a treatment regimen and it was employed in almost 20% of younger patients [9]. Compared with BU/CY (conv-MAC regimen), FLU/BU4 is less toxic and suppresses TRM. Moreover, the relapse rate is comparable between FLU/BU4 and BU/CY; therefore, FLU/BU4 is more efficacious than BU/CY for some types of patients [10].

FLU/BU4 should also be compared with TBI-based MAC regimens, which are still widely used in Japan, yielding strong anti-leukemic effects, even in chemotherapy sanctuary regions and guaranteeing engraftment by complete elimination of recipient immune effector cells [11]; thus, comparative data are needed for FLU/BU4 and conv-MAC, including TBI-based MAC regimens such as CY/TBI, when used as standard conditioning regimens for various types of AML patients.

Our data suggest that FLU/BU4 has similar anti-tumor effects not only to BU/CY, but also to CY/TBI and other intensive regimens. Moreover, our findings support the selection of FLU/BU4 even in younger patients, because major clinical outcomes (relapse, survival, TRM) are quite similar. Non-lethal regimen-related contraindications, including stomatitis, diarrhea, general fatigue, and nausea can be reduced in FLU/BU4 compared to conv-MACs [4]. More detailed subgroup analyses among younger patients should be performed in the future to identify characteristics of patients who can benefit from FLU/BU4 compared to conv-MAC regimens.

On the other hand, the results of this study, which focused on patients aged 50–59, revealed the superiority of FLU/BU4, compared with conv-MAC regimens, mainly because of lower incidence of TRM. This finding accords with that of the only other prospective trial comparing FLU/BU4 and BU/CY in CR patients, indicating that FLU/BU4 had lower 1-year TRM than BU/CY [4]. Lower incidence of lethal adverse effects of FLU/BU4 compared with conv-MACs may be due to the lower toxicity of FLU than CY (FLU/BU4 vs. BU/CY) or BU than TBI (FLU/BU4 vs. CY/TBI).

Due to missing data, we could not determine which cause was most associated with improved TRM in FLU/BU4. However, actual data on causes of death indicated that heart and/or lung failure was lower in the FLU/BU4 population in our analysis. This decrease of adverse events is more prominent in the elderly patient cohort, and a significant difference was observed in our sub-cohort of patients aged 50–59.

This confirmed improvement of TRM in FLU/BU4 was only observed in patients transplanted at CR status, but not non-CR status. Our analyses comparing CR vs. non-CR patients conditioned with FLU/BU4 revealed that increased incidence of cGVHD in the non-CR group may have resulted in worse TRM though not proven form the results of the study. A previous study indicated that “high” disease risk index (DRI) had only a weak association with aGVHD [12], whereas “very high” DRI (indicating stage risk is high) may be associated with SR-aGVHD [13], suggesting that disease control is pivotal to reduce the incidence of GVHD after transplant. Our results indicating non-CR status is a high risk for cGVHD and TRM are consistent with these studies, and suggest that obtaining CR at the time of HSCT is especially important. Recently available selective inhibitors (such as Bcl-2, FLT3 and IDH1/2) will produce more patients in CR status [14], and are expected to further reduce the risk of GVHD.

In summary, we compared FLU/BU4 vs. conv-MAC using the national registry in Japan, and our findings indicate that FLU/BU4 improved OS and TRM without increasing the relapse rate in AML patients, aged 50–59, transplanted at CR status. A FLU/BU4 regimen may be beneficial for such patients to improve outcomes and can be an optimal condition regimen [15]. Future analyses are necessary to validate our findings.

## Supplementary information

Table S1

## Data Availability

The datasets generated during and/or analyzed during the current study are available from the corresponding author on reasonable request.
