# Supplementary material for: Improved post-transplant outcomes for elderly acute myeloid leukemia patients conditioned with FLU/BU4 rather than conventional MAC regimens
Source: Bone Marrow Transplant. 2025 Apr 5;60(6):804–10. doi: 10.1038/s41409-025-02573-7 (PMC12151851; doi:10.1038/s41409-025-02573-7)
Supplement: Supplementary file 1 — Table S1 [file 41409_2025_2573_MOESM1_ESM.pdf]

Supplemental Table

Supplemental Table 1. Causes of death in CR patients aged 50 – 59

CR patients aged 50-59	FLU/BU4 (N=132)		conv-MAC (N=289)	
Relapse	42	31.8%	94	32.5%
TRM				
Infection	23	17.4%	50	17.3%
Viral	3	2.3%	5	1.7%
Bacterial	15	11.4%	26	9.0%
Fungal	2	1.5%	12	4.2%
Unknown	3	2.3%	7	2.4%
GVHD	12	9.1%	14	4.8%
aGVHD	7	5.3%	5	1.7%
cGVHD	5	3.8%	9	3.1%
Interstitial pneumoniae	18	13.6%	31	10.7%
CMV and PCP	2	1.5%	6	2.1%
Idiopathic	16	12.1%	25	8.7%
Organ failure				
Heart/Lung	5	3.8%	20	6.9%
Others	9	6.8%	14	4.8%
Graft failure	1	0.8%	6	2.1%
Bleeding	9	6.8%	8	2.8%
ARDS	3	2.3%	5	1.7%
TMA	1	0.8%	10	3.5%
VOD	5	3.8%	4	1.4%
Secondary malignancies	1	0.8%	7	2.4%
Other causes	3	2.3%	26	9.0%
Total	132	100.0%	289	100.0%

Abbreviations: aGVHD; acute graft versus host disease, cGVHD; chronic graft versus host disease, CMV; cytomegalovirus, PCP; pneumocystis pneumoniae, ARDS; acute respiratory distress syndrome, TMA; thrombotic microangiopathy, and VOD; veno-occulusive disase.
